# A Microfluidic Platform to design Multimodal PEG - crosslinked Hyaluronic Acid Nanoparticles (PEG-cHANPs) for diagnostic applications

**DOI:** 10.1038/s41598-020-63234-x

**Published:** 2020-04-07

**Authors:** Olimpia Tammaro, Angela Costagliola di Polidoro, Eugenia Romano, Paolo Antonio Netti, Enza Torino

**Affiliations:** 1 University of Naples Federico II, Department of Chemical, Materials and Production Engineering (DICMaPI), P.le Tecchio 80, 80125 Naples, Italy; 2 Fondazione Istituto Italiano di Tecnologia, IIT, Largo Barsanti e Matteucci 53, 80125 Naples, Italy; 3 Interdisciplinary Research Center on Biomaterials, CRIB, University of Naples Federico II, P.le Tecchio 80, 80125 Naples, Italy

**Keywords:** Medical imaging, Biomedical engineering, Microfluidics, Nanotechnology in cancer

## Abstract

The combination of different imaging modalities can allow obtaining simultaneously morphological and functional information providing a more accurate diagnosis. This advancement can be reached through the use of multimodal tracers, and nanotechnology-based solutions allow the simultaneous delivery of different diagnostic compounds moving a step towards their safe administration for multimodal imaging acquisition. Among different processes, nanoprecipitation is a consolidate method for the production of nanoparticles and its implementation in microfluidics can further improve the control over final product features accelerating its potential clinical translation. A Hydrodynamic Flow Focusing (HFF) approach is proposed to produce through a ONE-STEP process Multimodal Pegylated crosslinked Hyaluronic Acid NanoParticles (PEG-cHANPs). A monodisperse population of NPs with an average size of 140 nm is produced and Gd-DTPA and ATTO488 compounds are co-encapsulated, simultaneously. The results showed that the obtained multimodal nanoparticle could work as MRI/Optical imaging probe. Furthermore, under the Hydrodenticity effect, a boosting of the T1 values with respect to free Gd-DTPA is preserved.

## Introduction

Multimodal Imaging is a promising approach that allows the combination of different imaging techniques, overcoming limitations proper of every single modality^1,2^. For example, recently, Magnetic Resonance Imaging (MRI) and Optical imaging (OI) have been used in combination to obtain the excellent sensitivity of the OI with the high spatial resolution of the MRI^3–5^.

Image acquisition can occur at different times (asynchronously) requiring post-processing analyses performed through digital image manipulation techniques; however, the best consistency both in time and space is achieved when images are simultaneously acquired (synchronously)^6^. Despite the great advantages in the Hardware developments, probes able to support simultaneous acquisitions are still missing. Indeed, in current clinical practice, a cocktail of diagnostic compounds is injected with extremely high risk for the patient. In this scenario, the possibility to efficiently co-deliver through a single vector, different diagnostic compounds for different imaging modalities represents a key point. This challenge can be adequately tackled by applying nanotechnologies to the medical field^7–9^. Indeed, nanosystems can be used as vectors of active agents and their composition, size, shape, and surface chemistry can be finely modulated to obtain the simultaneous delivery of multiple diagnostic compounds with a significant impact on an early and accurate diagnosis^10–12^. Nanovectors can provide simultaneous visualization of the diseased site through different innovative imaging techniques, enhanced-circulation time for the diagnostic compounds, controlled release kinetics, and superior dose scheduling for improved patient compliance^13^. However, when systemically injected, nanoparticles are immediately sequestered by macrophages because of the opsonization, i.e. the formation of a corona of plasma proteins on the particle surface^14,15^. This phenomenon implies a reduced blood-circulation time and rapid clearance impairing the reaching of sufficient active agent concentrations at the diseased site. One of the most efficient ways to escape the macrophages capture and the protein corona formation is the covering of the particle surface with a hydrophilic material, such as Polyethylene glycol (PEG)^16–18^. In this way, it is possible to increase particle half-life and reduce the toxicity related to unspecific biodistribution^19,20^.

In the framework of the principles for the production of nanoparticles, nanoprecipitation-based methodologies open new possibilities regarding particle production, size regulation, active agent encapsulation and offer improvements in batch processes being less complicated and time consuming compared to other production techniques^19,21–26^.

Despite these advantages, limitations such as high batch-to-batch variations, non-homogenous reaction environment^27^ and insufficient production rate reveal the need to move towards techniques providing a higher control in the production of nanosystems with well-defined features.

In this sense, the advent of microfluidics constituted a breaking point. This simple, cost-effective and scalable technology poses as an alternative way to implement and consolidate production processes, strongly improving their controllability. Indeed, microfluidic-based production offers the possibility of tuning product composition and properties by process parameter adjustments^28–32^, such as flow rate ratio, polymer concentration, pH and temperature. In addition, when microfluidic platforms are used as microreactors, they provide improved space-time yields (product formed per reactor volume and time), producing faster reactions than bulk counterparts. Moreover, the degree of control over local environmental conditions is such to guarantee homogeneous products^33–36^.

The listed attributes elect microfluidics as a “disruptive technology” in tailorable nanovectors production and in the acceleration of their clinical translation^37,38^.

An efficient implementation of nanoprecipitation for particle production in microfluidics is the hydrodynamic flow focusing (HFF) regimen^39–42^. Indeed, as reported by Liu *et al*.^38^ the HFF can be used to improve different component mixing and to induce nanoprecipitation. In recent years, the literature showed a great interest in the use of HFF and effective mixing^28^. One of the first examples is presented by Karnik and co-workers^43^, who produced PLGA-PEG nanoparticles, studying the effect of the flow-rate ratio on particle size and polydispersity by micellization. Recently, Xu *et al*., proposed a continuous flow focusing–based strategy to improve the encapsulation efficiency (EE%) of Tamoxifen and Doxorubicin, a hydrophobic and a hydrophilic anticancer drug respectively, in PLGA NPs^27^. In this work, they demonstrated that an improved EE% translates into a more controlled drug release. Homogeneous drug distribution in the particle core is, in fact, able to avoid the initial burst release due to superficial drug distribution.

Recently, the continuous microfluidic method has also been used to develop more sophisticated multimodal nanoplatforms of very different materials. The most investigated combinations involve MRI/CT, PET/MRI, and Optical/MRI^5,6,44–46^. For example, regarding inorganic nanoparticles, Kim and co-workers^47^ presented nanocomposite microspheres incorporating alginate, iron oxide nanoclusters and Au nanorods for MRI/CT multimodal applications. In another work, using full biocompatible and already approved biomaterials, Russo *et al*.^48^. developed Hyaluronic acid nanoparticles crosslinked through divynilsulfone, encapsulating Gd-DTPA and ATTO633, as a new probe for MRI/Optical combination.

In the last example, Gd-DTPA, one of the most clinically relevant contrast agent for MRI^49,50^, was used without any chemical modification as approved for the clinical practice. However, its use has been limited over the years, because of a non-specific accumulation in kidneys and brain^51–54^. Many authors^55–57^ are working on the encapsulation of Gd-based CAs in nanosystems to reduce these side effects by exploiting a rational biodistribution. Additionally, Russo *et al*. demonstrated that when Gd-DTPA is encapsulated in polymeric networks, a complex equilibrium is formed by elastic stretches of polymer chains, water osmotic pressure and the hydration degree of Gd-CAs and that this equilibrium is able to boost the relaxivity of Gd-based Contrast Agents. This effect is called Hydrodenticity^58^. The ability of the Hydrodenticity to boost the relaxometric properties of Gd-chelates can, on one side, improve MRI performance and, on the other side, potentially reduce the administered dose of CAs keeping performances unmodified.

Starting from the evidence of the previous works related to the crosslinked Hyaluronic Acid NanoParticles (cHANPs)^41,48^ we are proposing an innovative process based on HFF to obtain in a ONE-STEP process the crosslinking reaction between thiolated Hyaluronic Acid (HA-SH) and Polyethylene glycol-vinyl sulfone (PEG-VS producing *pegylated* cHANPs (PEG-cHANPs), to produce multimodal probes for diagnostic applications.

Furthermore, the encapsulation of two diagnostic compounds, Gd-DTPA and ATTO 488, co-occurs, making these particles multifunctional and potentially suitable for multimodal MRI/ Optical diagnostics. In the combination of Optical and MRI imaging, it is possible to exploit 3D anatomical information and excellent spatial resolution provided by the MRI, and the high sensitivity and real-time analysis of the Optical imaging^9,59,60^.

The two probes encapsulation represents a potential for PEGylated crosslinked hyaluronic acid nanoparticles (PEG-cHANPs) theranostic applications since ATTO488 can be considered as the model of a low M_w_ drug.

## Results & Discussion

### Set-up of the microfluidic platform for PEG-cHANPs production

**Figure 1:**
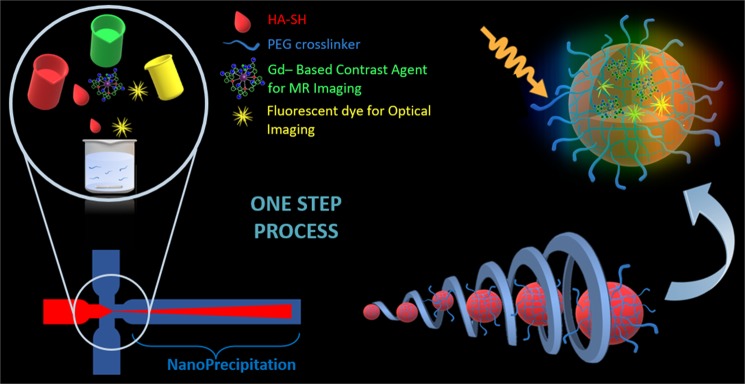
This schematic illustration presents the nanoprecipitation process implemented in microfluidics through an HFF approach for Gd-DTPA and ATTO 488 co-loaded PEG-cHANPs production. The process provides biocompatible and ready-to-use nanoprobes for Multimodal applications. This strategy condenses in a ONE-STEP process, a complex synthesis usually realized in batch mode through many different steps with significantly reduced controllability. The encapsulation of Gd-DTPA in the polymeric matrix provides a boosting of the MR signal in accordance with the Hydrodenticity theory.

Hydrodynamic Flow-Focusing (HFF) nanoprecipitation is a consolidate method for the production of nanoparticles. The regimen of hydrodynamic flow focusing ensures uniform reaction conditions and particle formation kinetics. A study about the feasibility of the particle production process is conducted to evaluate the effect of the flow rate ratio on the obtaining of the nanoparticle and their properties such as composition, size, shape and superficial charge. Moreover, optimized conditions for the microfluidic translation of the nucleophilic attack reaction are evaluated both in terms of total polymer concentration and of molar ratio between functional groups (SH/VS) Fig. 1.

**Figure 2:**
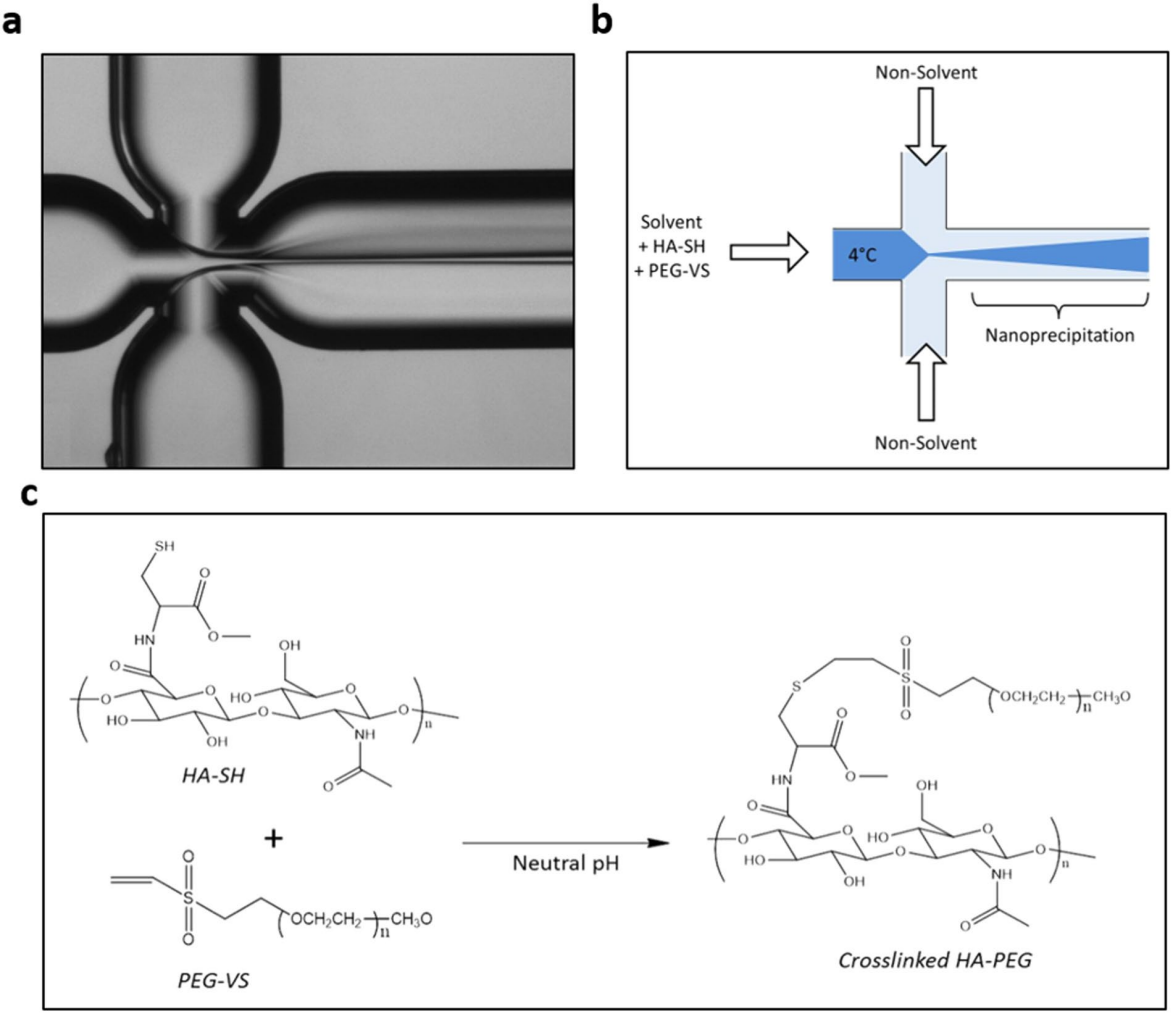
Schematic illustration of Microfluidic experimental set-up. (**a**) Optical Fluorescence Microscopy Image of Flow-Focusing pattern; (**b**) Qualitative Illustration of crosslinking strategies processed in our microfluidic device; (**c**) Crosslinking reaction of HA thiol groups with the terminal double bond of PEG-VS.

The nanoprecipitation process is implemented in a microfluidic chip with an X-junction configuration (“Droplet - Junction Chip”, depth x width: 190 µm x 390 µm) where particle formation occurs by diffusion and nanoprecipitation (Fig. 2a). The middle channel is injected with an aqueous solution composed of thiolated hyaluronic acid (HA-SH) and polyethylene glycol- vinyl sulfone (PEG-VS); the side channels are injected with pure acetone to provide the extraction of the water phase (Fig. 2b).

The production process involves the simultaneous control of many steps. The two different polymers are nanoprecipitated and, in the meanwhile, the Michael addition reaction between thiol groups and vynilsulfone moieties starts to produce at the same time crosslinked and pegylated nanoparticles (Fig. 2c). In batch processes, the only driving force for reaction occurrence and group interaction is thermal motion. In a flow focusing regimen, the middle stream is squeezed by lateral flows to a width related to the flow rate ratio (FR^2^), defined as the ratio between the volumetric flow rate of the solvent phase (middle channel) and the non-solvent phase (side channels)^41^. Thus, tuning the FR^2^, it is possible to reduce the τ_mix_ of the components improving the probability of reaction occurrence^28^. This effect confers to the HFF approach the accurate control over final nanoparticle properties by simple tuning of focused stream width and process parameter adjustment.

### Feasibility study: Flow Rate Ratio effect

**Figure 3:**
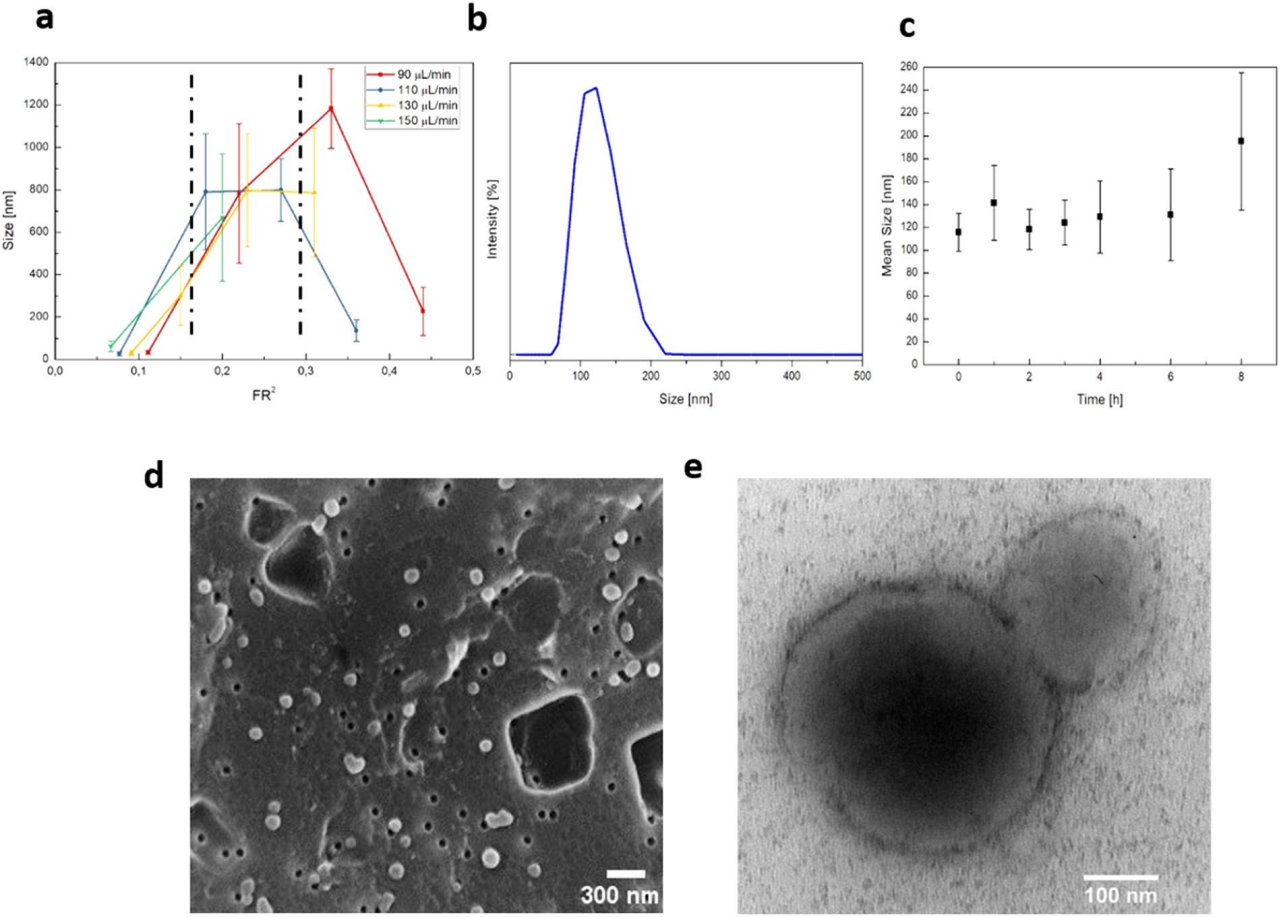
Study of morphological and platform properties. (**a**) Study of flow rate ratio (FR^2^) effect on nanoparticles size; (**b**) DLS profile of cross-linked nanoparticles obtained with standard conditions; (**c**) Swelling behavior of crosslinked nanoparticles at different time point until 8 hours; (**d**) FE-SEM image of NPs, scale bar 300 nm; (**e**) TEM image, scale bar 100 nm, of cross-linked hyaluronic acid nanoparticles with PEG-vinyl sulfone in water. Abbreviations: FE-SEM, Field emission scanning electron microscope; TEM, Transmission electron microscope; DLS, Dynamic light scattering.

In the previous section, we defined the platform set-up in terms of chip geometry and operative parameters. To characterize the resulting particle production in terms of size and morphology, the effect of the Flow Rate Ratio (*FR*^2^) is studied. In our work, different *FR*^2^ values ranging from 0.06 to 0.44 are tested to characterize their influence on nanoprecipitation phases of nucleation and growth. Middle channel flow rates are varied from 10 µL/min to 40 µL/min while side ones from 90 µL/min to 160 µL/min (further details in supplementary data Table S.1 and Fig. S1). In order to avoid flow rate fluctuations and to guarantee flux stability, each *FR*^2^ value is tested singularly, without variations during the process. Some combinations could not be explored because of middle channel backflow or massive precipitation. In Fig. 3a the change of particle size in function of *FR*^2^ is shown. All data of Z-Average and standard deviation are obtained by DLS measurements, morphologically analyzed by SEM and plotted fixing the side channel flow rate. Each measurement has been repeated at least three times.

The plot shows that there is an increasing trend of particle size with *FR*^2^ values, ranging from 30 nm to 800 nm. Variability in mean particle size and standard deviation can be attributed not only to the *FR*^2^ variation but also to the complexity of simultaneous phenomena taking place. In detail, we identified three different regions. In the first one, *FR*^2^ is lower than 0.12 and the mean particle size is around 30 nm. In the second region, *FR*^2^ ranges from 0.12 to 0.27 and the particle size increases from ~ 250 nm to ~ 800 nm. Finally, the third region with *FR*^2^ higher than 0.27 is characterized by massive precipitation. Indeed, in this range, SEM observations show the formation of big aggregates and films of material that compromise the DLS analysis. This result suggests that increasing the *FR*^2^ over a certain limit, the production moves towards a region in which every variation would not produce any effect different from uncontrolled and massive aggregate precipitation. At the same time, moving towards the lowest values of *FR*^2^ there is the production of very small particles that can provide a low loading capability and could not be optimal for our final goal of simultaneous encapsulation of different probes. For these reasons, we selected as Operative-Working Region (OR) for FR^2^ the second region where the variation of FR^2^ from 0.12 to 0.27 corresponds to a size variation between 250 nm and 800 nm. Moreover, since a *FR*^2^ value of 0.27 (obtained at 30 µL/min – solvent flow rate – and at 110 µL/min – non-solvent flow rate) provides a fine and stable flow focusing and absence of precipitates in the mixing channel, it is selected as the “*standard condition*” and used in the next experiments. In each trial, both HA-SH and PEG-VS are dissolved in water and injected in the middle channel. However, the main need is to guarantee that the Michael addition takes place in the focused stream in a controlled fashion avoiding its premature occurrence in the preparation step or before the reaching of the nozzle. To this purpose, the effect of the temperature on the reaction rate is studied. Two different strategies, Low Temperature (LT) and Room Temperature (RT) are compared. In the first case, the temperature of the injected polymer solution is kept at 4 °C both during the preparation step and injection; in the second one, both the mixing and processing of the solution occurred at room temperature. For all cases, no temperature control on the chip is present. However, we assume that, for the RT strategy, the mixing Temperature in the main channel is constant while for the LT strategy a Temperature gradient moving toward the RT is obtained, achieving the favorable conditions for the promotion of the crosslinking reaction. It is worthy of notice that, also at RT, the solvent solution is processed with no difficulties, meaning that the crosslinking is not completed before injection. However, results show that in this case, a film of material is formed, and the particle formation is impaired. When the temperature is kept at 4 °C a uniform population of nanoparticles is obtained (further details in supplementary data Fig. S2). These observations reveal that Temperature control is necessary to slow down the reaction rate and to control its occurrence in the device. To study the effect of the Temperature, all trials are conducted in a *standard condition* of 30 µL/min for the middle channel and 110 µL/min for the side ones.

### Michael addition reaction optimization

A study of the influence of the functional group ratio (defined as the number of thiol groups over the number of vinyl sulfone groups, SH/VS) on particle size and the crosslinking occurrence, is conducted. Literature data on the SH/VS ratio are reporting a value of 1.2 and a total polymer concentration of 6% w/V as the optimized condition for batch processes^61–63^.

To make a comparison about the traditional batch processes and the more efficient microfluidic approach and to evaluate the impact of this ratio on the stability of the PEG-cHANPs, a study about the microfluidic implementation of the Michael addition is conducted. At our standard *FR*^2^ value of 0.27, the total polymer concentration is reduced up to 0.23% w/V and significantly smaller values of the functional group ratio are explored. SH/VS value has been varied from 0.0009 to 0.2. It is worth noticing that the similar SH/VS ratios of 0.0011 and 0.0009 have a relevant difference that is due to the difference between the substitution degree of the compounds. The thiolated Hyaluronic Acid (HA-SH) has a very low substitution degree of only 5%, while the PEG-VS is linearly functionalized (100%). This significant chemical difference produces a great change in the concentration of the injected total solution, even though there is only a small variation in the SH/VS ratios. It is particularly true in a microfluidic environment where a slight change in the concentration could promote a relevant change in the fluidodynamics and, in our particular case, in the thermodynamics of the nanoprecipitation. In details, the SH/VS ratio of 0.0009 is obtained with HA-SH concentration constant at 0.1% w/V and PEG-VS concentration of 0.223% w/v, in greater excess of PEG-VS with respect to the concentration used for SH/VS of 0.0011 (0.18% w/V). As a consequence, we observed polydisperse precipitation due to the formation of unstable PEG-VS substructures. The optimized condition is found at SH/VS value of 0.0011 that is three order of magnitude lower than the literature reference. In these conditions, a uniform population of spherical particles with a mean dimension of 140 nm is obtained (Fig. 3b).

All the other explored values show that the mean particle size increases with increasing SH/VS values. The DLS distribution presented in Fig. 3b, points out the significant role of this parameter in regulating particle size and properties, when comparing the mean particle size variation with SH/VS ratio to the set of DLS data reported in Fig. S1g, h and i.

The effectiveness of the crosslinking reaction is indirectly characterized through a study of particle stability in water since in absence of reaction a fast and irreversible swelling of the nanoparticles would be observed (further details in supplementary data Figs. S3–S5). The particle swelling behavior has been studied for 8 hours through DLS measurements. Figure 3c shows that there is no significant variation in the mean particle size and polydispersity up to 6 hrs, from 6 to 8 hr a slight increase in the size is observed. Particle morphology has been characterized by SEM and TEM (Fig. 3d,e). The surface charge of these particles is measured through a Z-potential measurement. As expected from the combination of two negatively charged polymers^64,65^, it reveals a superficial charge of −28 ± 5 mV.

### Simultaneous encapsulation of Gd-DTPA and ATTO 488 and Loading Capability of the PEG-cHANPs

Previous optimizations led to the definition of the following conditions as *standard conditions* for the particle production: T = 4 °C, FR^2^ = 0.27 and SH/VS = 0.0011. Starting from these conditions, the simultaneous encapsulation of Gd-DTPA and ATTO 488 is tested to provide multifunctional properties to the nanovectors. Briefly, the payload agents are dissolved in the polymer solution before the injection. Later, the nanoprecipitation occurs in the mixing channel producing the encapsulation of the compounds. We have observed that the presence of these two agents is not significantly influencing the stability of the flow focusing on the microfluidic device even if higher precipitation at the interface between the solvent and the non-solvent phase in the mixing channel has been visually observed. DLS and SEM analyses show a slight increase of the mean size at 150 ± 25 nm (further details in supplementary data Fig. S6).

ICP-MS analyses have been used to quantify the amount of Gd-DTPA entrapped in nanoparticles. Gd-loaded PEG-cHANPs exhibit a loading capability (LC) of 60%. In the case of PEG-cHANPs coloaded with Gd-DTPA and ATTO 488, LC related to the Gd-DTPA is showing a slight decrease down to 25%. (further details in supplementary data Table S2).

The amount of co-loaded ATTO 488 has been determined through measurement with Multiplate Reader Photometer and the effective quantity is calculated starting from a calibration curve (see supplementary data Fig. S7). Results show a LC of 60%. In perspective, the encapsulation of the fluorescent compound can be used as a model for a possible drug encapsulation to provide a theranostic function to the system.

### *In-vitro* T_1_

**Figure 4:**
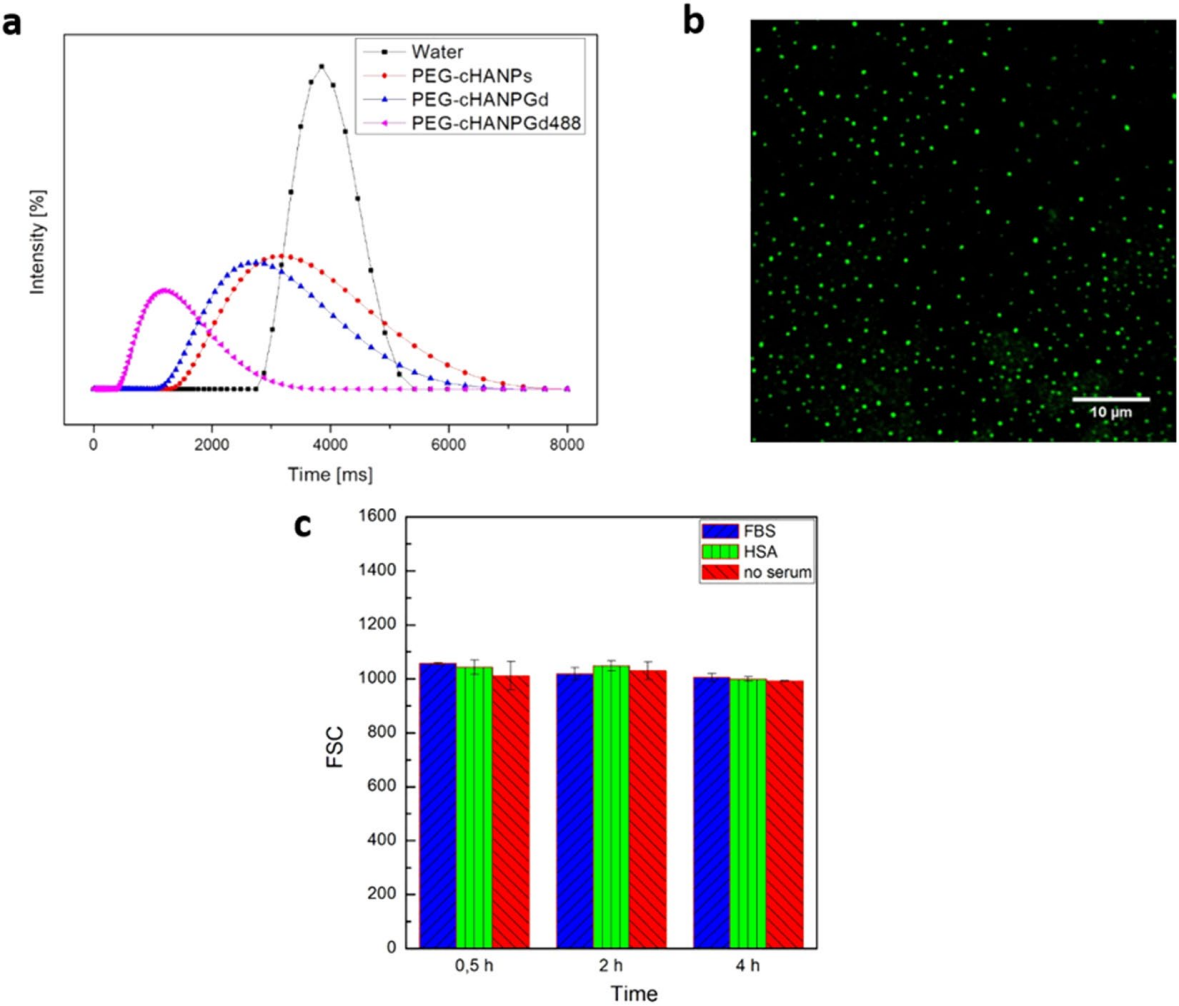
Characterization of functionalized multifunctional nanoparticles: (**a**) *In vitro* MR. Comparison of longitudinal relaxation time distributions of water (-■-), empty PEG-cHANPs suspension (-●-), PEG-cHANPs loaded with Gd-DTPA (-▲-); PEG-cHANPs co-loaded with ATTO488 and Gd-DTPA (-◀-); (**b**) Optical Imaging of co-loaded PEG-cHANPs under Confocal Microscopy (acquisition spectra 500–530 nm), scale bar 10 μm; (**c**) *In vitro* cytotoxicity: FSC signal of co-loaded-PEG-cHANPs in contact with U87 MG cells in three different serum condition at three different time points. Abbreviation: FSC, forward scattering.

*In-vitro* longitudinal relaxation time T_1_ evaluation is performed for pure water as control, empty PEG-cHANPs, PEG-cHANPs loaded with Gd-DTPA and PEG-cHANPs co-loaded with Gd-DTPA and ATTO 488. The longitudinal relaxation time T_1_ is measured at 37 °C and at 1.5 T. Each measurement shows a reduced mean T_1_ value with respect to pure water Fig. 4a.

Moreover, the relaxation time of free Gd-DTPA at different molar concentrations is measured in order to compare this value to the relaxation time obtained from different nanoparticle formulations (a plot of concentration VS relaxivity in supplementary data Fig. S8). The comparison reveals that T_1_ = 1640 ms associated with Gd loaded PEG-cHANPs corresponds to an equivalent free-Gd-DTPA content of 135 µM, a value slightly higher than the effective concentration inside our nanosystems (110 µM by ICP-MS measurement), anyway 1.22 times higher than the free Gd-DTPA at the same concentration. Interestingly, Gd-DTPA and ATTO 488 co-loaded PEG-cHANPs analysis reveal a T_1_ = 1130 ms, 5 times higher than the T1 signal associated with the same concentration of free Gd-DTPA. However, both Gd loaded and Gd-ATTO coloaded PEG-cHANPs confirm the boosting of the Relaxometric properties of the metal chelate and, therefore, also this new formulation is preserving the effect of Hydrodenticity^58^. To explain the synergic effect of the Dye and Gd-chelate on the T_1_ values, we hypothesize that in the presence of the hydrophilic ATTO488, the hydration of the hydrogel nanoparticles is increased because of dye molecule solvation. Indeed, by definition, in the process of solvation, molecules are surrounded by concentric shells of solvent^66^. It can directly affect the mobility of water molecules that are coordinated around Gd-DTPA^67^.

### Confocal microscopy

Confocal microscopy observations are performed to assess ATTO 488 encapsulation. The presence of spherical fluorescent spots confirms the encapsulation of the dye in spherical nanoparticles Fig. 3b. However, nanoparticles size cannot be evaluated due to the low spatial resolution of the optical light.

### Preliminary *in-vitro* cell tests

PEG-cHANPs *in-vitro* cytotoxicity is preliminarily studied realizing particle contacts with U87 MG cells. Measurements are performed at different time points and in different serum conditions in order to study the role of PEG in protein corona formation: Fetal Bovine Serum (FBS), Human Serum Albumin (HSA), and no serum. DLS analyses show no significant changes in the mean particle size, confirming nanoparticle stability at 37 °C in each serum condition up to 6 hrs (further details in the Supplementary data Fig. S9). Flow cytometer analyses are used to measure the Forward Scattering (FSC), a parameter representative of cell size and consequently used as an indirect indication of cell viability^68^.

The FSC plot in time shows that no significant changes in the number of live cells are present in any condition Fig. 3c. Indeed, the contact of NPs with cells is affecting FCS signal neither in different sera nor at different time points. Thus, we can hypothesize that in the *in-vitro* environment, PEG is conferring to particles a stealth property that could reduce the formation of a protein corona. It can also be concluded that particles are not exerting a toxic effect on cells.

### Conclusions

Microfluidics is considered a disruptive technology for pharmaceutical manufacturing and is becoming a gold standard in the production of nanoparticles for drug delivery. Here, a ONE-STEP microfluidic synthesis of *pegylated* crosslinked Hyaluronic Acid NanoParticles (PEG-cHANPs) has been investigated for the production of multimodal nanoparticle for potential Multimodal Imaging application. A feasibility study has been conducted exploiting all the process parameters such as FR^2^, Temperature, and SH/VS ratio. Results have shown the ability of the microfluidic platform to tune the properties and control the size of the particles from 30 nm up to 800 nm. Moreover, it has been proved that the reaction conditions in the microfluidic device are less prohibitive than in a batch mode; indeed, we are able to stabilize the nanoparticles at an SH/VS ratio several orders of magnitude lower than the value reported in the literature. These conditions are able to guarantee particle stability in water. Furthermore, the proposed strategy allows to co-encapsulate in a one-step process two different diagnostic compounds, Gd-DTPA and ATTO 488, to produce a probe for Multimodal applications. The designed probe is able to boost the MR signal thanks to the unique effect of the Hydrodenticity, furthermore amplifying the T1 up to 5 times in presence of the ATTO 488. This result could potentially lead to a reduction of the administered dose of CA safekeeping high quality images.

Finally, due to the versatility of the technology, this microfluidic platform can pave the way for the fast and accurate synthesis of advanced probes for theranostic applications.

## Materials & Methods

Hyaluronate Thiol (HA-SH) with 5 mol % substitution, MW 50 kDa, and mPEG-Vinylsulfone (PEG-VS), MW 2 kDa, were purchased from Creative PEGWorks. Diethylenetriaminepentaacetic acid gadolinium (III) dihydrogen salt hydrate Gd-DTPA (97%), Ethanol (ACS reagent, (200 proof), absolute; Acetone (puriss. p.a., ACS reagent, reag. ISO, Ph. Eur., ≥ 99.5%); Sodium Hydroxide NaOH (ACS reagent, ≥ 97.0%, MW 40.00 g/mol), ATTO 488 MW 804 g/mol (Ex/Em 488/520) were purchased by Sigma-Aldrich Co. Dulbecco Modified Eagle Medium high glucose (DMEM), Fetal Bovine Serum (FBS), Phosphate Buffer Saline (PBS) and Human Serum Albumin (HSA) for cell *in-vitro* study were purchased by Sigma Aldrich Co. Water for synthesis and characterization, was purified by distillation, deionization, and reserve osmosis (Milli-Q Plus; Merck, Darmstadt, Germany).

### Microfluidics set-up for Flow Focusing (FF) approach

A quartz microfluidic device “Droplet - Junction Chip” (depth x width: 190 µm x 390 µm), purchased from Dolomite Centre Ltd, was used. The internal surface of the channels is coated by a hydrophobic material. On the chip, there are two separate droplet junctions, which can be used in combination. For all the experiments, an X-junction with three inlets and a single outlet channel has been used. The device has a flow-focusing geometry with a 90° angle between the inlets to enhance the diffusion process Fig. 2b. It is compatible with Chip interface H for fluidics connections. Three-way isolation ethylene tetrafluoroethylene (ETFE) valves, connected to the syringes and the microfluidic device, make the automatic fill-in of the syringes feasible, thus allowing a continuous dispensing of reagents. The linkage between fluorinated ethylene propylene tubes and the device is carried out through a specially designed connection with polytetrafluoroethylene connectors. The glass syringes (volume 2.5–5 mL) are controlled by a pressure pump (Low-Pressure Syringe Pump neMESYS 290 N by CETONI). The flow focusing regimen in the microchannel is observed through an optical fluorescence microscope (Olympus IX71) with a 4x scanning objective. A schematic representation is presented in Fig. 2a.

### Synthesis in one-step process of PEGylated Hyaluronic Acid Nanoparticles

Different flow rates were tested and the influence of the flow rate ratio variation on the particle production, size and morphology are determined. For the feasibility study, an aqueous solution of thiolated HA (HA-SH) at a concentration ranging from 0.01 to 0.5% w/V is tested. The initial solution is kept under continuous stirring for at least 15 min and then PEG-Vinyl sulfone (PEG-VS) (ranging from 0.005 to 0.2% w/v) is added. This solution is injected in the middle channel with flow rates ranging from 10 µL/min to 40 µL/min; acetone, used as non-solvent, is injected laterally varying flow rates from 90 µL/min to 160 µL/min in order to induce nanoprecipitation in a flow focusing approach. Precipitated NPs are collected in a glass Petri dish containing about 25 mL of non-solvent phase.

In our microfluidic system, a study about the reagent concentration and the influence of the functional group ratio on the nucleophilic addition of the carbanion (HA-SH) to the unsaturated carbonyl compound (PEG-VS) is performed. Each successful experiment is repeated at least ten times.

### Purification Recovery and Characterization of nanoparticles

Purification is performed by dialysis using a solvent gradient across the Spectra Por Cellulose Membrane 6 (Molecular Weight Cut Off, MWCO 50000 Da). A typical procedure consists of loading collected samples into dialysis tubing and keeping the external solution under continuous stirring at 200 rpm to increase the diffusional rate. It is worth highlighting that all nanoparticles are purified in ethanol to remove partially the unreacted reagents.

The first purification step is in ethanol:

 The sample is purified for 1 h in 70% Acetone +30% Ethanol; 1 h in 50% Acetone + 50% Ethanol; 1 h in 30% Acetone + 70% Ethanol; 2 h in 100% Ethanol.

The second purification step is in water:

The sample is purified for 1 h in 70% Ethanol +30% MilliQ water; 1 h in 50% Ethanol + 50% MilliQ water; 1 h in 30% Ethanol + 70% MilliQ water; 2 h in 100% MilliQ water.

Dynamic light scattering (DLS) is used to determine nanoparticle size, polydispersity and superficial charge (Zetasizer Nano ZS, Malvern UK). The wavelength of the laser is 633 nm and the scattering used is 173°. 1 mL of sample is put in 12 mm square glass cuvettes for 90° sizing (Optical Cuvette, Sarstedt). Zeta potential measurements are also performed at a temperature of 25 °C on a Zetasizer Nano ZS (Malvern, UK), fitted with a high-concentration zeta potential cell. Characterization of the particle morphology and structure is realized through electron microscopy. For TEM analysis (Cryo-TEM TECNAI by FEI) 20 µL of the purified sample are dropped on a Carbon Film membrane (Agar scientific) and dried before use. SEM observations (Carl Zeiss Ultraplus Field Emission) are made depositing 200 µL of purified samples on polycarbonate Isopore membrane filters (different cut-offs 0.05, 0.1 and 0.2 µm) through an ultrafiltration vacuum system. On the membrane filter 7 nm of Au are deposited.

### Simultaneous encapsulation of Gd-DTPA and ATTO 488

ATTO 488 and Gd-DTPA are added to the HA-SH aqueous solution at a concentration of 10 nmol/mL and 0.1% w/V respectively. Then, the solution is mixed with the crosslinker PEG-VS.

The loading capability of Gd-DTPA is calculated by Inductively Coupled Plasma (ICP-MS) NexION 350 measurements. All data are collected and processed using the Syngistix Nano Application Module. Gd-DTPA is measured at m/z 157 using a 100 μs dwell time with no settling time. The concentration of loaded fluorophore is determined using Multiplate Reader Photometer (Enspire Perkin-Elmer) (λ _ex/em_ 488–500 nm). The calibration curve is set in the range of 0–0.2 nmol/ml, to avoid system saturation with higher concentration. To overcome the scattering due to the nanoscale size of particles, all measurements are conducted with a dilution ratio of 1:4.

### *In vitro* T_1_

*In vitro* MR has been realized both on empty and on differently loaded NPs and results were compared to control water solutions at a known concentration of Gd-DTPA. After vigorous stirring, 300 μl of the sample are put in glass tubes and changes in relaxation time (T_1_) were evaluated at 1.5 Tesla by Minispec Bench Top Relaxometer (Bruker Corporation). The relaxation time distribution is obtained by a CONTIN Algorithm and the relaxation spectrum is normalized by its processing parameters. The integral of a peak corresponds to the contribution of the species exhibiting this peculiar relaxation to the relaxation time spectrum. Experiments were repeated at least ten times.

### Confocal Microscopy

The optical properties of the loaded NPs are tested by confocal microscopy (Leica Microsystems TCS SP5 Laser Scanning Confocal Microscopy). Different preparation protocols are used to characterize fluorescent NPs. They are observed both in solution, in a Willco-dish glass or on a polycarbonate Isopore membrane.

### Swelling behavior of the NPs

PEG-cHANPs swelling behavior is studied to indirectly characterize reaction occurrence and efficiency. The solution was observed 0, 1, 2, 3, 4, 6 and 8 hours from water dialysis to identify possible variation in size.

### Preliminary *in-vitro* cell tests

Studies of NPs cytotoxicity on Human brain Glioblastoma astrocytoma cells (U87-MG) are conducted to preliminary asses their biocompatibility.

NPs internalization is analyzed after their incubation with live cells through a CyFlow Space (Sysmex Partec) flow cytometer. A 488 nm wavelength laser is used to excite NPs and fluorescence is collected using a 595–660 nm channel. U87 MG cells are seeded in 12-well plates (2 × 10^5^ cells/well) and incubated for 24 h before NPs addition. Subsequently, cells are incubated with culture medium DMEM, 1% penicillin/streptomycin and 1% L-glutamine supplied with NPs (50 µg/mL) in three different serum conditions: FBS (10% V/V), HSA (20% V/V) and no serum. They are observed after 30 minutes, 2 and 4 hours of contact. At different time points, the medium is removed, and the samples are washed three times with PBS (1×) to ensure the removal of non-internalized particles. Every flow cytometry analysis is conducted at least in triplicate after cell detachment by trypsinization.

## Supplementary information

Supplementary Data.
