# Author Correction: A Microfluidic Platform to design Multimodal PEG - crosslinked Hyaluronic Acid Nanoparticles (PEG-cHANPs) for diagnostic applications

**DOI:** 10.1038/s41598-020-67066-7

**Published:** 2020-06-12

**Authors:** Olimpia Tammaro, Angela Costagliola di Polidoro, Eugenia Romano, Paolo Antonio Netti, Enza Torino

**Affiliations:** 1 University of Naples Federico II, Department of Chemical, Materials and Production Engineering (DICMaPI), P.le Tecchio 80, 80125 Naples, Italy; 2 Fondazione Istituto Italiano di Tecnologia, IIT, Largo Barsanti e Matteucci 53, 80125 Naples, Italy; 3 Interdisciplinary Research Center on Biomaterials, CRIB, University of Naples Federico II, P.le Tecchio 80, 80125 Naples, Italy

Correction to: *Scientific Reports* 10.1038/s41598-020-63234-x, published online 07 April 2020

The Acknowledgements section in this Article was omitted. The Acknowledgments section should read:

“This work has been supported by the project financed by the MIUR Progetti di Ricerca di Rilevante Interesse Nazionale (PRIN) Bando 2017 - grant 2017MHJJ55.”

