# Supplementary material for: A Microfluidic Platform to design Multimodal PEG - crosslinked Hyaluronic Acid Nanoparticles (PEG-cHANPs) for diagnostic applications
Source: Sci Rep. 2020 Apr 7;10:6028. doi: 10.1038/s41598-020-63234-x (PMC7138812; doi:10.1038/s41598-020-63234-x)
Supplement: Supplementary file 1 — Supplementary Data. [file 41598_2020_63234_MOESM1_ESM.docx]

# **TITLE**

A Microfluidic Platform to design Multimodal PEG - crosslinked Hyaluronic Acid Nanoparticles (PEG-cHANPs) for diagnostic applications

Olimpia Tammaro^1,2^§, Angela Costagliola di Polidoro^1,2^§, Eugenia Romano^1,2^, Paolo Antonio Netti^1,2,3^, Enza Torino^1,2,3^*

^1^ University of Naples Federico II, Department of Chemical Engineering, Materials and Industrial Production, P.le Tecchio 80, 80125, Naples, Italy;

^2^ Istituto Italiano di Tecnologia, IIT - Center for Advanced Biomaterials for Health Care, CABHC@CRIB, Largo Barsanti e Matteucci, 80125, Naples, Italy;

^3^ Interdisciplinary Research Center on Biomaterials, CRIB, University of Naples Federico II, P.le Tecchio 80, 80125, Naples, Italy

§ Olimpia Tammaro and Angela Costagliola di Polidoro contributed equally to this work

*corresponding author: Enza Torino - [enza.torino@unina.it](mailto:enza.torino@unina.it)

# SUPPLEMENTARY DATA

## Flow rate ratio effect:

Different combinations of middle channel flow rate and side channel flow rate are explored. Some combinations cannot be explored because of backflow or focusing instability.

Table S 1: Size analysis with DLS for the combinations of examined FR^2^ collected in three different regions

*Not Available (NA) is referred to trials that cannot be completed

| **Region** | **Middle channel FR [µL/min]** | **Side channel FR [µL/min]** | **FR^2^** | **Mean Size** | **PDI** |
| --- | --- | --- | --- | --- | --- |
| 1^st^ | 10 | 160 | 0,0625 | NA* | NA* |
|  |  | 150 | 0,06666667 | 62,43 | 0,319 |
|  |  | 130 | 0,07692308 | 30,88 | 0,25 |
|  |  | 110 | 0,09090909 | 27,61 | 0,25 |
|  |  | 90 | 0,11111111 | 33,71 | 0,129 |
|  | 20 | 160 | 0,125 | NA* | NA* |
| 2^nd^ | 20 | 150 | 0,13333333 | NA* | NA* |
|  |  | 130 | 0,15384615 | 301 | 0,425 |
|  |  | 110 | 0,18181818 | 791 | 0,239 |
|  | 30 | 160 | 0,1875 | NA* | NA* |
|  |  | 150 | 0,2 | 670 | 0,4 |
|  | 20 | 90 | 0,22222222 | 783 | 0,355 |
|  | 30 | 130 | 0,23076923 | 798 | 0,223 |
|  |  | 110 | 0,27272727 | 800 | 0,068 |
| 3^rd^ | 40 | 130 | 0,30769231 | 787 | 0,3 |
|  | 30 | 90 | 0,33333333 | 1184 | 0,05 |
|  | 40 | 110 | 0,36363636 | 135,8 | 0,28 |
|  |  | 90 | 0,44444444 | 227,5 | 0,5 |

b)

c)

a)


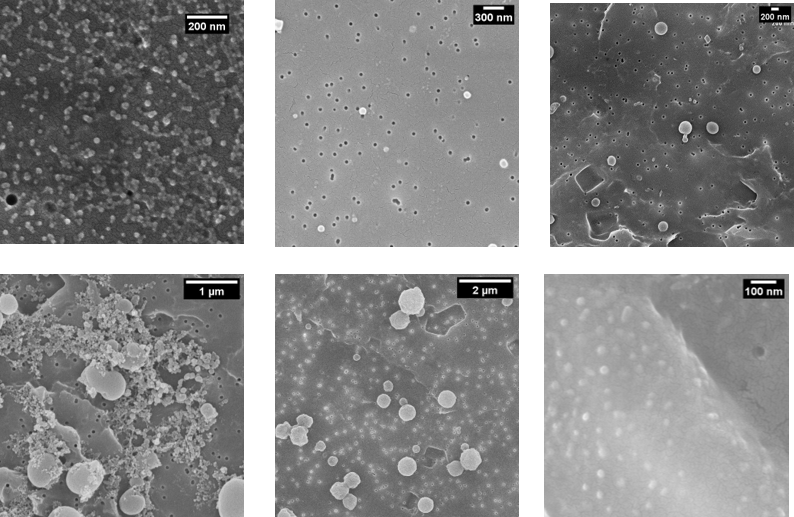


d)

e)

f)


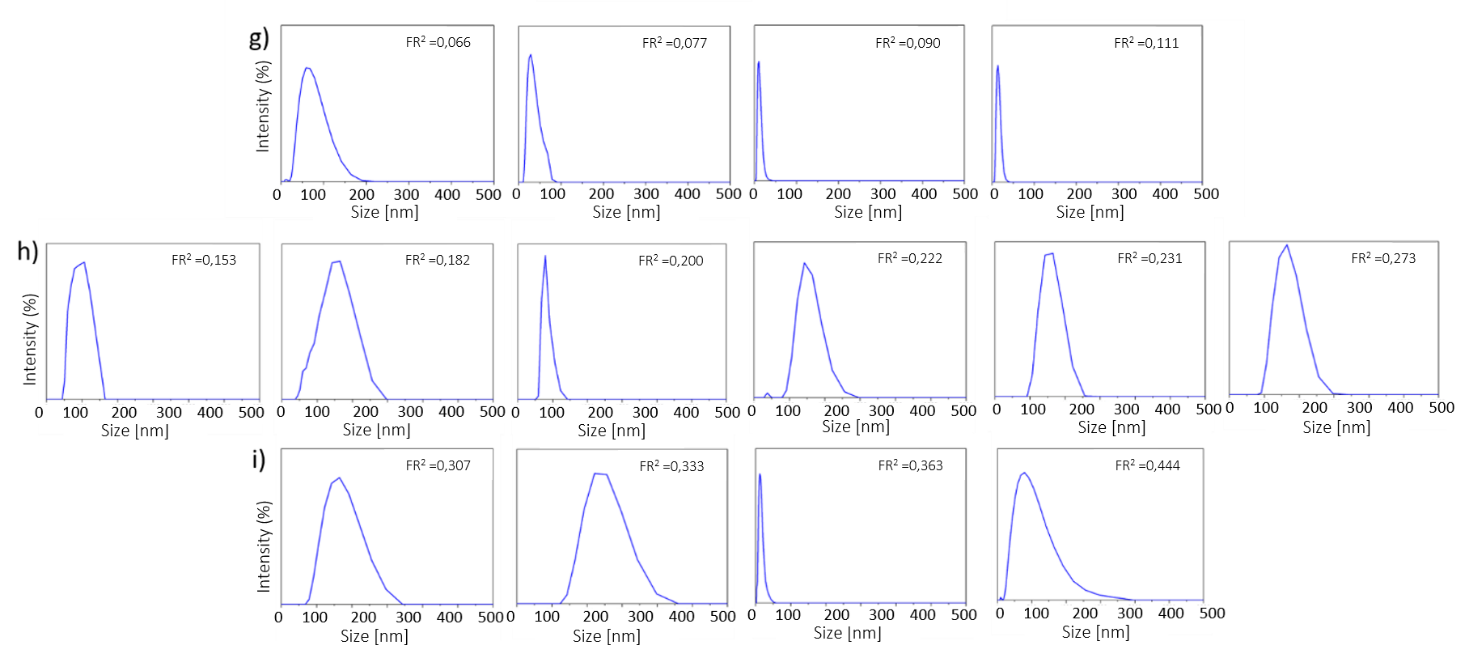
Figure S 1: SEM images and DLS distributions presenting particle size and morphology variation with FR^2^. a) First FR^2^ region, b-c-d-e) Second FR^2^ region, f) Third FR^2^ region, g) First region size distributions, h) Operative Working Region (OR) size distributions, i) Third region size distribution where DLS data are not representative of the real particle size as from SEM images

## Temperature effect

The study of the effect of temperature on particle production is conducted by testing different temperatures for the middle channel solution injection. Herein, SEM images for the injection at 4°C (Fig S2.a) and at Room Temperature (Fig S2.b) are reported.

a)

b)


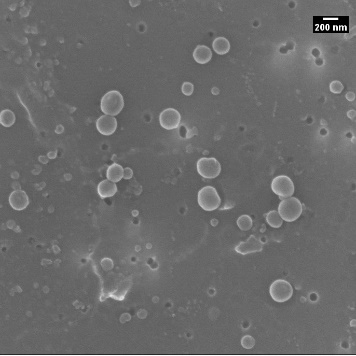

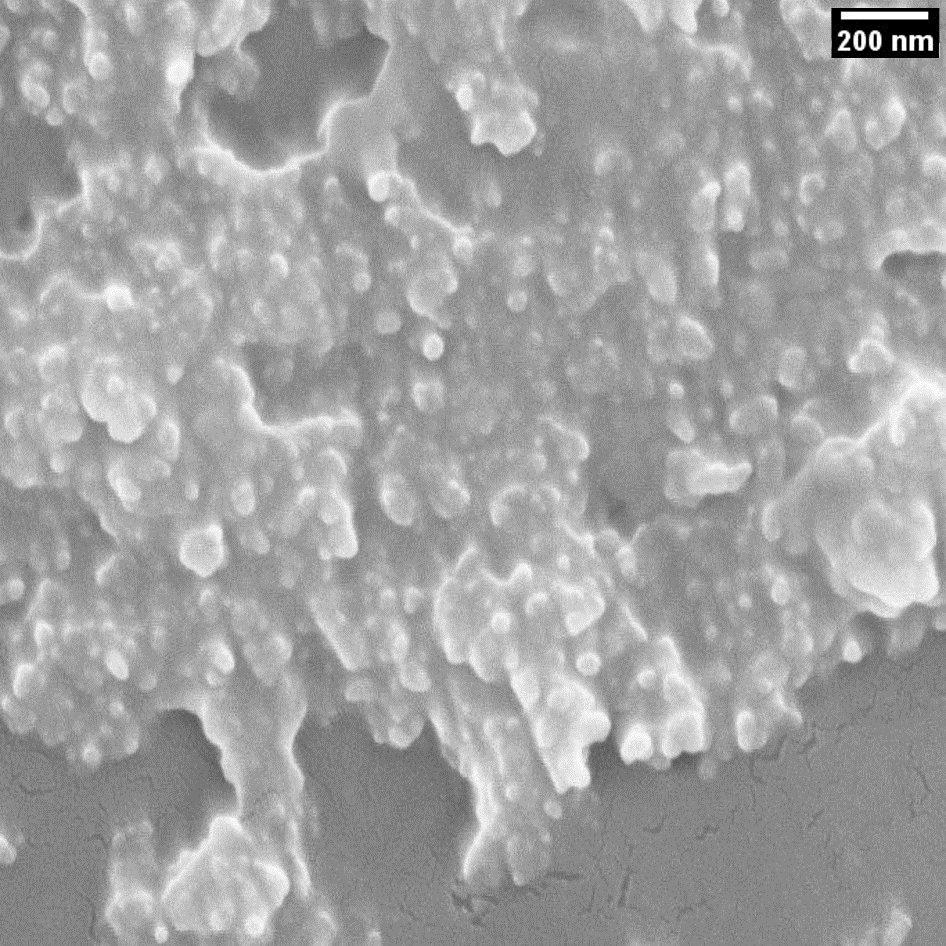


Figure S 2: Temperature study: SEM images of (a) 4°C and (b) Room temperature solution injection

As clear from images, when no temperature control is present a film of material is formed and the particle formation impaired. However, no difficulties are present in the injection of the room temperature solution, meaning that the gelation is not completed and that the reaction is just started in the syringe.

## SH/VS molar ratio effect

Different functional group molar ratios are tested, and the reaction occurrence is evaluated. SEM images show how strong is the influence of this parameter on particle size, morphology and polydispersity.


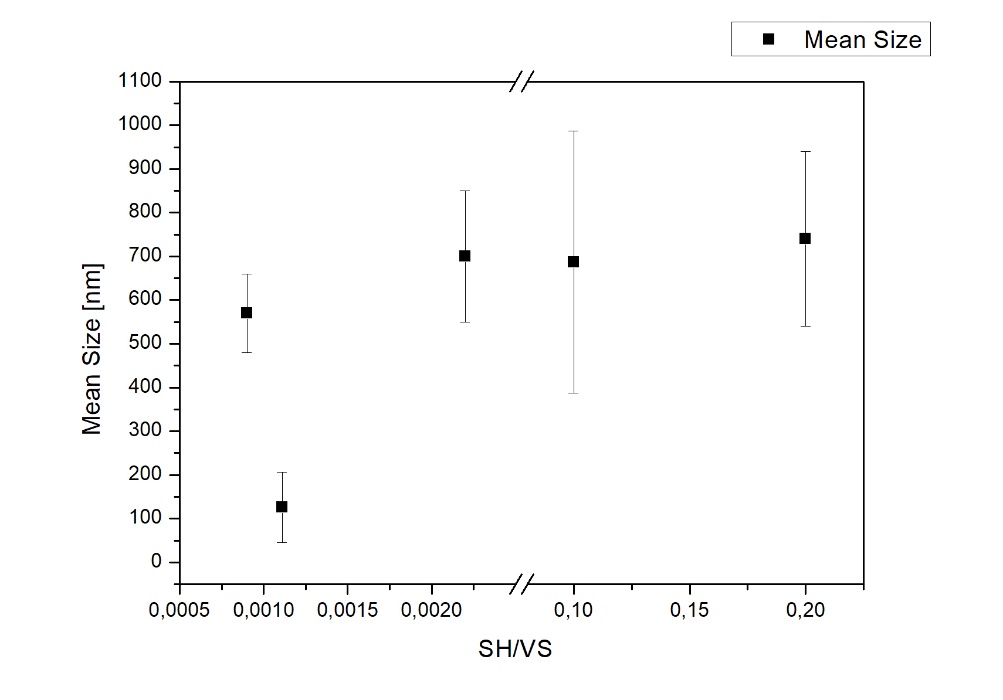


Figure S 3: DLS measurement: Particle size and polydispersity variation with SH/VS ratio.


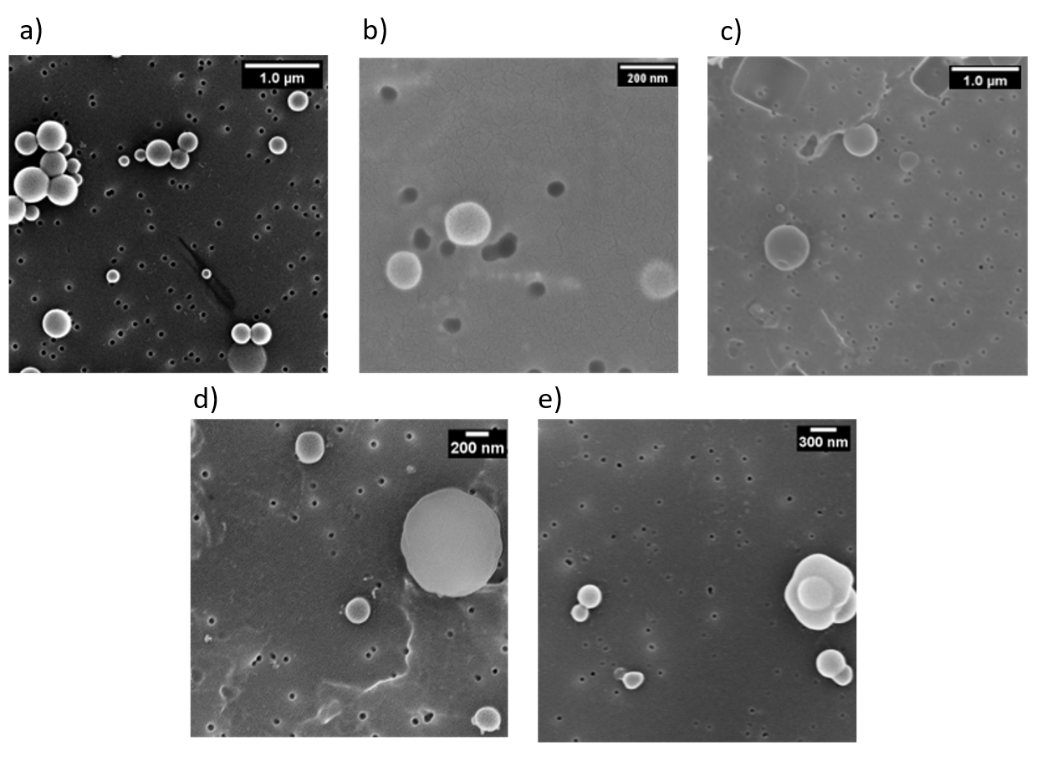


Figure S 4: SEM images of (a) SH/VS = 0,0009 (b) SH/VS = 0,0011 (c) SH/VS = 0,0022 (d) SH/VS = 0,1 (e) SH/VS = 0,2.


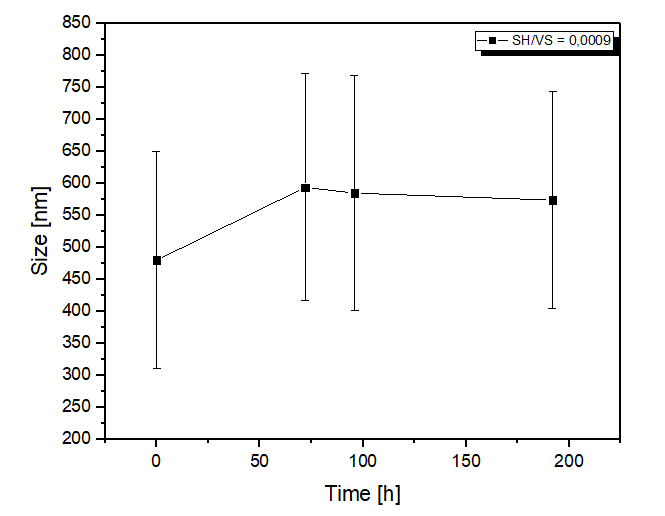


Figure S 5: DLS measurements: a stability study of SH/VS=0,0009 in standard FR^2^=0,27

## Encapsulation data

DLS measurements show that particle size is slightly affected by Gd-DTPA and Atto 488 encapsulation. Empty and loaded PEG-cHANPs exhibit a very similar size (Fig. S6).


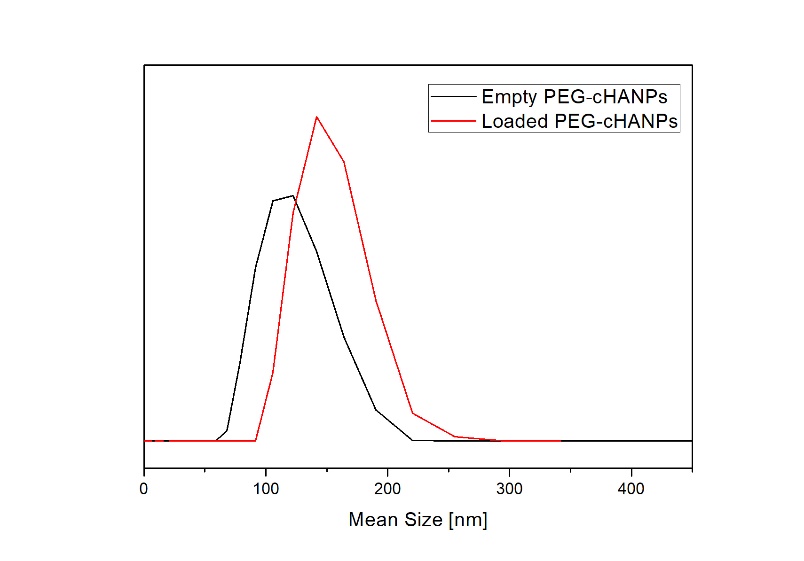


Figure S 6: DLS measurements of Empty and Loaded PEG-cHANPs

Gd-DTPA concentration is evaluated by ICP-MS analysis, results are presented in Table S2.

Table S 2: ICP-MS Gd-DTPA molar concentration within nanoparticles

|  | Experimental Concentration | Theoretical Concentration | EE % |
| --- | --- | --- | --- |
| GD LOADED PEG-ChanpS | 0,000110 | 0,000187 | 60% |
| GD & ATTO CO-LOADED PEG-cHANPS | 0,000048 | 0,000187 | 25% |

The molar concentration of ATTO 488 encapsulated is determined by a Multiplate Reader Photometer (Enspire Perkin-Elmer) (λ _ex/em_ 488-500 nm). The calibration curve is set in the range of 0–0,2 nmol/ml, to avoid system saturation with higher concentrations. The calibration curve has an R^2^ value of 0,9959 (Fig. S7). Measurements on samples show a value of fluorescence intensity of 350 ±30, which corresponds to a concentration of 0,6 nmol/mL (considered the dilution ratio of 1:4). This value allowed us to define for ATTO 488 an EE% of 60%.


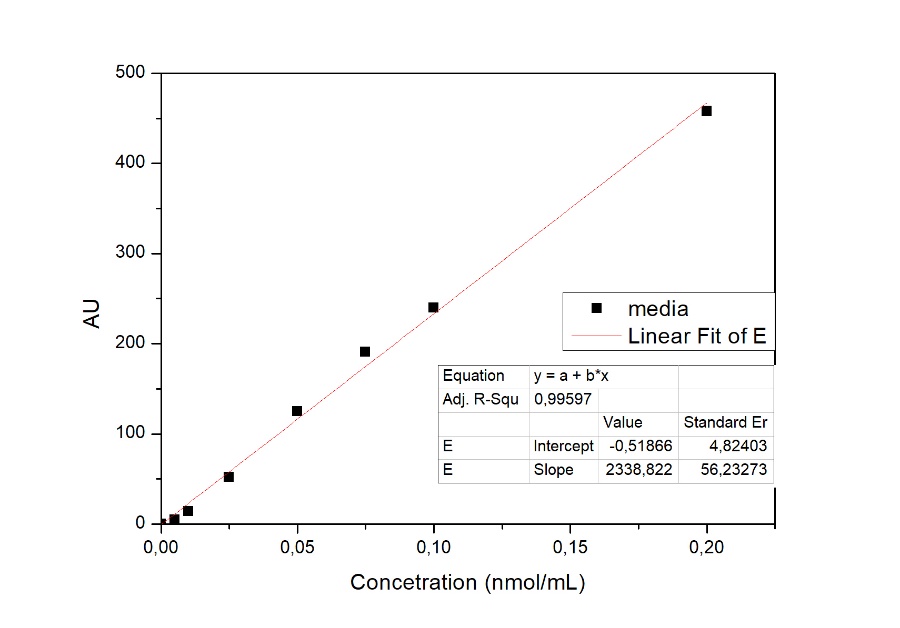


Figure S7: Calibration Curve from Multiplate Reader Photometer for ATTO488 content

Gd-DTPA concentrations evaluated by ICP-MS are then compared to the longitudinal relaxation time T_1_ of free Gd-DTPA. A curve of relaxivity VS concentration is reported (Fig. S8).


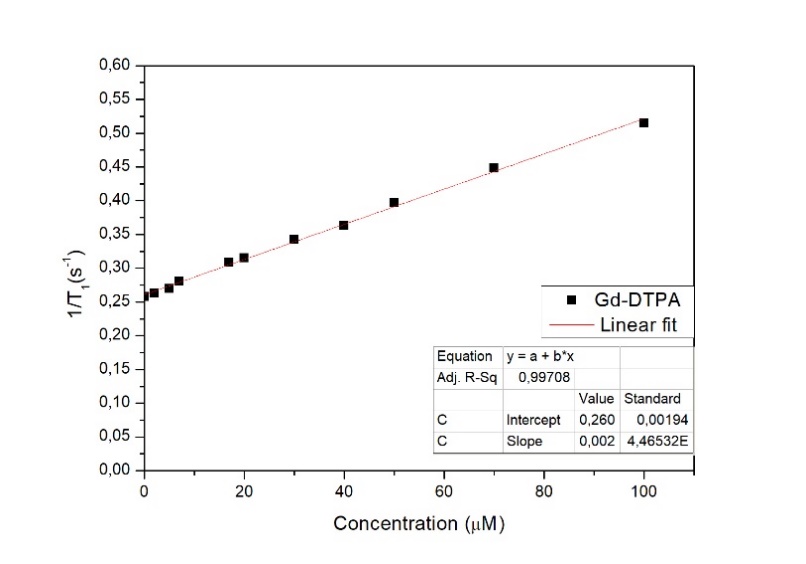


Figure S 8: Calibration Curve of relaxivity from Minispec Measurement for Gd-DTPA content

Particle relaxation times are compared to the data obtained from this curve. Elaborations show an influence of the polymeric matrix on the CA relaxation time, in accordance with the Hydrodenticity.

## Preliminary in-vitro cell tests

A stability study of nanoparticles at 37°C, in different serum conditions and at defined time points is presented.


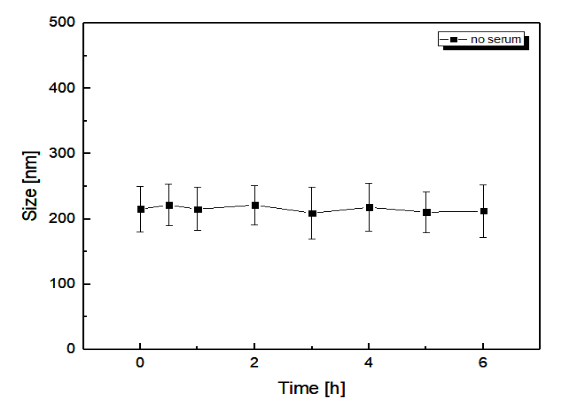

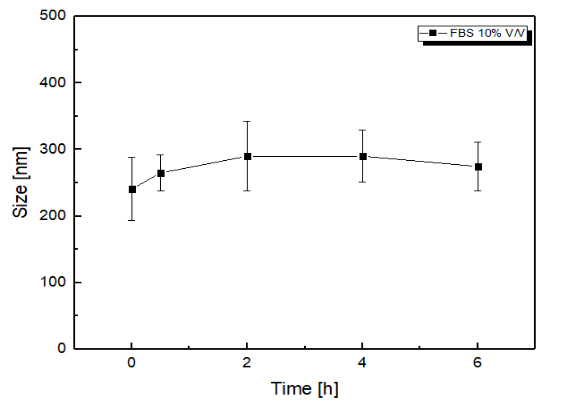


b)

a)

c)


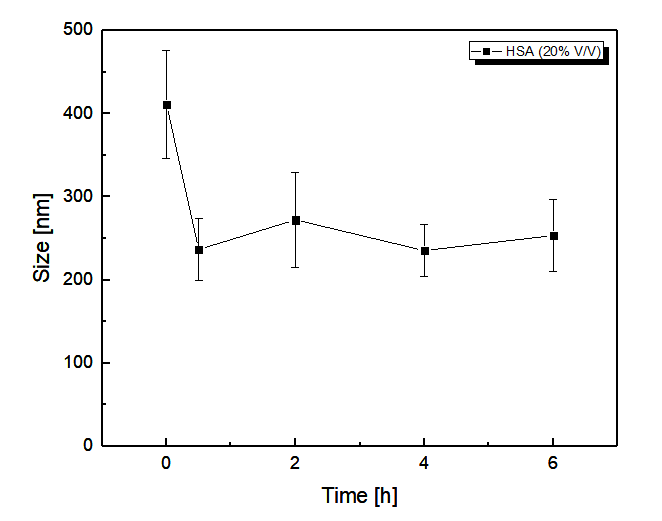


*Figure S9: Stability Study of ATTO loaded PEG-cHANPs at 37°C in different serum conditions. (a) no serum (b) FBS 10% V/V (c) HSA 20% V/V*
